# Pertussis Toxin Is a Robust and Selective Inhibitor of High Grade Glioma Cell Migration and Invasion

**DOI:** 10.1371/journal.pone.0168418

**Published:** 2016-12-15

**Authors:** Andrew S. Gilder, Lei Wang, Letizia Natali, Nicki Karimi-Mostowfi, Coralie Brifault, Steven L. Gonias

**Affiliations:** 1 Department of Pathology, University of California San Diego, La Jolla CA, United States of America; 2 Department of Histology and Embryology, Xuzhou Medical University, Xuzhou, Jiangsu, China; 3 Department of Pharmacy, University of Pisa, Pisa, Italy; Tel-Aviv University, ISRAEL

## Abstract

In high grade glioma (HGG), extensive tumor cell infiltration of normal brain typically precludes identifying effective margins for surgical resection or irradiation. Pertussis toxin (PT) is a multimeric complex that inactivates diverse G_i/o_ G-protein coupled receptors (GPCRs). Despite the broad continuum of regulatory events controlled by GPCRs, PT may be applicable as a therapeutic. We have shown that the urokinase receptor (uPAR) is a major driver of HGG cell migration. uPAR-initiated cell-signaling requires a G_i/o_ GPCR, N-formyl Peptide Receptor 2 (FPR2), as an essential co-receptor and is thus, PT-sensitive. Herein, we show that PT robustly inhibits migration of three separate HGG-like cell lines that express a mutated form of the EGF Receptor (EGFR), EGFRvIII, which is constitutively active. PT also almost completely blocked the ability of HGG cells to invade Matrigel. In the equivalent concentration range (0.01–1.0 μg/mL), PT had no effect on cell survival and only affected proliferation of one cell line. Neutralization of EGFRvIII expression in HGG cells, which is known to activate uPAR-initiated cell-signaling, promoted HGG cell migration. The increase in HGG cell migration, induced by EGFRvIII neutralization, was entirely blocked by silencing FPR2 gene expression or by treating the cells with PT. When U87MG HGG cells were cultured as suspended neurospheres in serum-free, growth factor-supplemented medium, uPAR expression was increased. HGG cells isolated from neurospheres migrated through Transwell membranes without loss of cell contacts; this process was inhibited by PT by >90%. PT also inhibited expression of vimentin by HGG cells; vimentin is associated with epithelial-mesenchymal transition and worsened prognosis. We conclude that PT may function as a selective inhibitor of HGG cell migration and invasion.

## Introduction

Pertussis toxin (PT) is a multimeric protein complex formed by assembly of five distinct subunits into a hexamer [1]. After gaining entrance into eukaryotic cells, the PT S1 subunit expresses enzymatic activity, catalyzing ADP ribosylation of target proteins [1, 2]. The most important targets for PT S1 subunit are α subunits of G_i/o_ hetero-trimeric G proteins [1–3]. α subunit modification uncouples diverse G protein-coupled receptors (GPCRs) from their effector systems accounting for most of the activities of PT.

Because numerous GPCRs are PT-sensitive, the effects of PT on cell physiology are cell type- and context-dependent. PT inhibits cell migration by diverse mechanisms, including but not limited to the disabling of chemokine receptors such as CCR2, CCR5, and CX_3_CR1 [4–6] and inhibiting the response to lysophosphatidic acid [7,8]. We have shown that, in high grade gliomas (HGG), including glioblastoma, the urokinase receptor (uPAR) can function as a major driver of cell migration, especially in cells that have been treated with therapeutics that target the EGF Receptor (EGFR) [9, 10]. uPAR is a glycosylphosphatidylinositol-anchored membrane protein and thus not directly affected by PT; however, the function of uPAR in cell signaling requires the PT-sensitive GPCR, N-formyl Peptide Receptor 2 (FPR2), as an essential co-receptor [11, 12].

Unlike many malignancies, HGGs are lethal due to local invasion as opposed to metastasis, and the invasion pattern is highly irregular, precluding complete surgical margins or well-defined areas for irradiation [13]. Identifying novel approaches for controlling HGG cell migration and invasion is therefore an important objective. A number of studies have examined the potential to exploit PT as a therapeutic. In preclinical rodent model systems, systemically administered PT has demonstrated efficacy in counteracting hypertension [14]. PT was effective against tumors in a C6 glioma model and in an RG2 glioma model in combination with temozolomide [15, 16]. Signs of toxicity that might preclude further testing of PT were not reported. PT also has been applied into the bladders of patients with bladder cancer without local or systemic toxicity [17]. Prompted by the known role of PT in blocking uPAR-initiated cell-signaling [11] and the effects of uPAR on HGG cell migration [9], we undertook studies to test whether PT inhibits the aggressiveness of HGG cells.

In HGGs, EGFR gene amplification is common and the EGFR may be mutated to form a derivative, called EGFRvIII, which signals constitutively in the absence of ligand [18–20]. To model HGGs in which EGFR signaling is activated, we studied a series of HGG-like cell lines that express EGFRvIII. Herein, we show that PT, at doses up to 1.0 μg/mL, has little or no effect of HGG cell viability or proliferation. However, in studies with three distinct HGG-like cell lines, PT substantially inhibited HGG cell migration and invasion through Matrigel. PT also down-regulated expression of vimentin, which is a biomarker of epithelial-mesenchymal transition (EMT) expressed by motile HGG cells and associated with a negative prognosis [21]. The activity of PT in inhibiting HGG cell migration and invasion suggests a novel approach for treating HGG.

## Materials and Methods

### Cell Lines and Reagents

HGG cells were cultured in Dulbecco’s Modified Eagles Medium (DMEM) supplemented with 10% fetal bovine serum (FBS), unless otherwise noted. U251 cells, formerly known as U373 cells, and Ink4a/Arf^-/-^ astrocytes, both of which express EGFRvIII, are previously described [22, 23]. In the U251 cells (U251vIII), EGFRvIII expression was controlled by a doxycycline-repressible promoter [10]. These cells were maintained in the absence of doxycycline unless otherwise indicated. To neutralize EGFRvIII expression in U251vIII cells, the cells were cultured in the presence of 1.0 μg/mL doxycycline for 5 days, as previously described [10]. Wild-type EGFR-over-expressing U251 cells are previously described [22]. EGFRvIII-expressing U87MG (U87vIII) cells also are previously described [20].

Primary mouse astrocyte cultures were prepared from mixed mouse cortical cell preparations [24]. Briefly, cerebral cortices from newborn C57BL6/J mice were dissected and carefully stripped of their meninges. Intact cortices were mechanically and enzymatically dissociated using the Neural Tissue Dissociation Kit (Miltenyi Biotec). Mixed glial cultures were established in DMEM-F12 medium (1:1) supplemented with GlutaMAX^TM^ (Life Technologies), 10% FBS, and 100 U/ml Fungizon® (Life Technologies). After one week of culture, microglia and oligodendrocyte precursor cells were harvested by shaking the mixed cultures on an orbital shaker at 200 rpm for 30 min and then at 240 rpm for 4 h. The remaining confluent astrocyte layer was detached by trypsinization and plated in T75 culture flasks in astrocyte culture medium consisting of DMEM high glucose (Hyclone), 10% FBS and 100 U/ml Fungizon®. Cell migration studies were performed 14 days later.

PT was from Millipore. Mouse laminin was from Invitrogen. Phalloidin conjugated to Alexa-488, basic fibroblast growth factor (bFGF), ProLong Gold Antifade with DAPI, and B27 Supplement were from Life Technologies. EGF was from Sigma. EGFR-specific antibody was from EMD Millipore. Antibodies that recognize EGFR phospho-Tyr-1068, vimentin, Sox-2, and β-Actin were from Cell Signaling Technology. uPAR-specific antibody was from R&D Systems.

### Neurosphere Culture

U87vIII cells were cultured as free floating neurospheres in non-coated sterile petri dishes. Neurosphere cell culture medium contained DMEM-F12 glutaMAX (Thermo-Fisher Scientific) supplemented with B27, 20 ng/mL bFGF, and 20 ng/mL EGF. Neurospheres were allowed to form for 6 days before performing experiments. At that time, neurospheres reached a maximum diameter of 60–100 μm.

### FPR2 Gene-silencing

U251vIII cells were seeded at a density of 30,000 cells/cm^2^ and transfected with 75 nM human FPR2-targeting siRNA (Thermo, ID4150) or non-targeting control (NTC) siRNA (ON-TARGET*plus* non-targeting pool, Thermo) using lipofectamine 2000 according to the manufacturer’s protocol. The transfection was allowed to proceed for 20 h before seeding cells for Transwell cell migration assays.

### HGG Cell Migration

Cell migration was studied *in vitro* using Transwell chambers with 8 μm pore-containing membranes (Corning) according to the manufacturer’s instructions. Adherent monolayer cultures were dissociated and seeded into Transwells at a density of 10,000 cells/unit. Transwell membranes were pre-coated on both surfaces with 1.0 μg/mL vitronectin. FBS (1%) was added to the lower chamber as a chemoattractant. PT was added to both chambers as indicated. Migration was allowed to proceed for 18 h. Transwell membranes were stained using Diff-Quick and were mounted onto slides for counting. A Leica DM2500 light microscope equipped with a Leica DFC420 digital camera and Leica Application Suite software was used to acquire representative images and the number of migrated cells was quantified using Image-J software (NIH). Within each experiment, each set up conditions was studied in triplicate.

To examine migration of HGG cells isolated from suspended neurospheres, Transwell membranes were coated on both surfaces with 10 μg/mL laminin. An equal number of cells, including approximately 50 multicellular neurospheres greater than 75 μm in mean diameter, were transferred without disruption into the upper chamber of each Transwell. Migration was allowed to proceed for 4 h. Cell migration was assessed as described above.

### HGG Invasion of Matrigel

HGG cell invasion through Matrigel was studied using preformed Corning Matrigel Invasion Transwell systems, as suggested by the manufacturer. FBS (1%) was added to the bottom chamber. PT was added to both chambers as indicated. Cells were allowed to invade for 40 h. The number of cells that penetrated through the Matrigel and to the lower membrane surface was determined as described in our cell migration experiments.

### Cell Survival and Proliferation Assay

WST-1 proliferation agent (Sigma) was used to identify and quantitate viable HGG cells. Cells were plated at a density of 10,000 cells per well in 12 well plates and cultured in the presence of 0.01–1.0 μg/mL PT or vehicle, in medium with 10% FBS or in serum-free medium (SFM). At the indicated times, WST-1 reagent was incubated with the cells for 1 h. WST-1 cleavage was determined by measuring absorbance at 450 nm using a Spectramax M2 scanning multiwall spectrophotometer.

### Immunoblot Analysis

Cells were extracted in RIPA buffer (20 mM sodium phosphate, 150 mM NaCl, pH 7.4, 1% Triton X-100, 0.5% sodium deoxycholate and 0.1% SDS) supplemented with EDTA-free protease inhibitor cocktail (Thermo Scientific) and 1 mM sodium orthovanadate. Protein load was normalized using bicinchoninic acid assay. For SDS-PAGE, 30 μg of cellular protein was loaded per well. Proteins were electrotransferred to PVDF membranes and incubated with primary antibodies followed by horseradish peroxidase-conjugated secondary antibody (Jackson Immuno-Research). Bands were imaged using Super Signal West Pico substrate or Femto substrate (Thermo Fisher Scientific).

### Immunofluorescence (IF) Microscopy

U251vIII cells were seeded onto CC2-coated chamber slides (Nunc) and treated with 1.0 μg/mL PT or with vehicle for 24 h in medium supplemented with 1.0% FBS. Cells were then fixed in 4% paraformaldehyde and permeabilized for 5 min with 0.1% Triton-X-100. Slides were blocked with 0.5% donkey serum and then probed with vimentin-specific antibody, followed by Alexa-488-conjugated secondary antibody, or stained with Alexa-488-conjugated phalloidin. Slides were mounted using ProLong Gold Antifade with DAPI and images were collected using the 40x objective on a Leica DMIRE2 fluorescence microscope equipped with a Hamamatsu digital camera with SimplePCI software.

### Confocal Imaging of Neurospheres

U87vIII cells were allowed to form neurospheres for 6 days. The cells were then fixed in 4% paraformaldehyde and permeabilized with ice cold acetone for 10 min. The cells were pre-incubated with 0.5% goat serum and stained with Rhodamine-phalloidin and DAPI. The neurospheres were examined by fluorescence microscopy using an Olympus FV1000 confocal microscope. Images were analyzed using the Fluoview FV10-ASW software package and are presented as Z-projections of 49 slices.

### Statistics

Studies were analyzed by Student's t-test or by one way ANOVA with a Tukey’s *post hoc* analysis, using GraphPad Prism 5 (GraphPad Software). The following significance parameters were used: *, p< 0.05; **, p< 0.01; ***, p< 0.001.

## Results

### PT Inhibits HGG Cell Migration

To examine the effects of PT on HGG cell migration, we used Transwell cell migration units. To model HGGs in which EGFR signaling is activated, as is frequently the case in patients with HGG [18–20], we studied cell lines that express EGFRvIII. EGFRvIII is constitutively active in the absence of ligand; however, its enzymatic activity is attenuated compared with EGF-ligated wild-type EGFR [25]. The three cell lines studied here express uPAR, a major driver of HGG cell migration [9, 11, 26]. uPAR-activated cell-signaling and cell migration require FPR2, which is a known PT target [11, 12].

**Fig 1:**
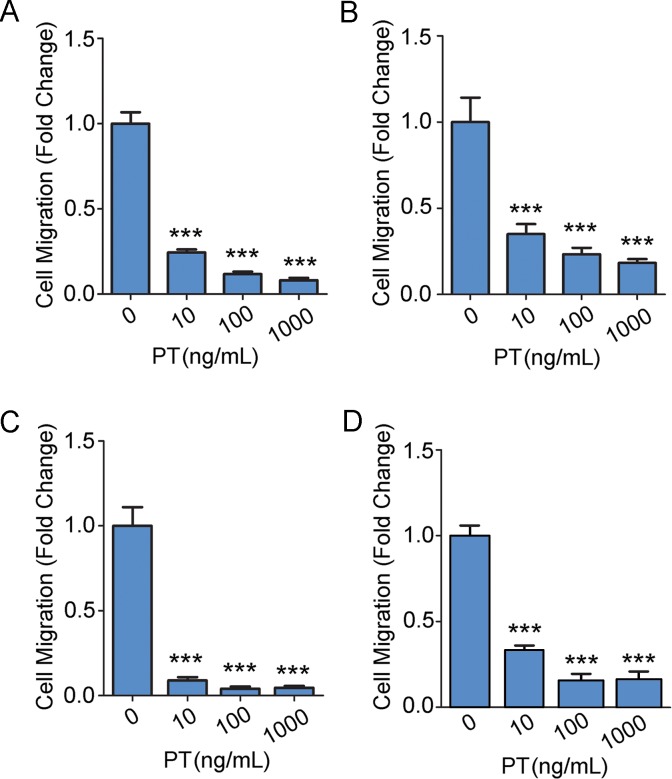
PT inhibits HGG cell migration. (A) U251vIII cells were allowed to migrate in Transwells for 18 h. PT was added to both Transwell chambers at the indicated concentrations. Migration is shown relative to untreated controls (mean ± SEM; n = 3; ***p<0.001). (B) Migration was performed as described in panel A with U87vIII cells (mean ± SEM; n = 3; ***p<0.001). (C) Migration was performed as described in panel A with Ink4a/Arf^-/-^vIII astrocytes (mean ± SEM; n = 3; ***p<0.001). (D) Migration was performed as described in panel A with mouse cortical astrocytes (mean ± SEM; n = 2 with three internal replicates; ***p<0.001).

Fig 1A shows that PT inhibited migration of U251vIII cells. The effects of PT on U251vIII cell migration were dose-dependent and highly significant (p<0.001) even at 10 ng/mL. As a second cell culture model system, we studied U87vIII cells. Once again, cell migration was inhibited by PT in a dose-dependent manner (Fig 1B). Fig 1C shows that PT robustly inhibited migration of EGFRvIII-expressing Ink4a/Arf^-/-^ astrocytes (Ink4a/Arf^-/-^vIII cells), which also are HGG-like cells [26].

As a control, we examined normal astrocytes isolated from mouse cerebral cortex and propagated in primary culture [24]. PT significantly inhibited migration of these cells (Fig 1D), indicating that the PT-sensitive pathways, involved in the control of HGG cell migration, are not unique to cancer cells.

**Fig 2:**
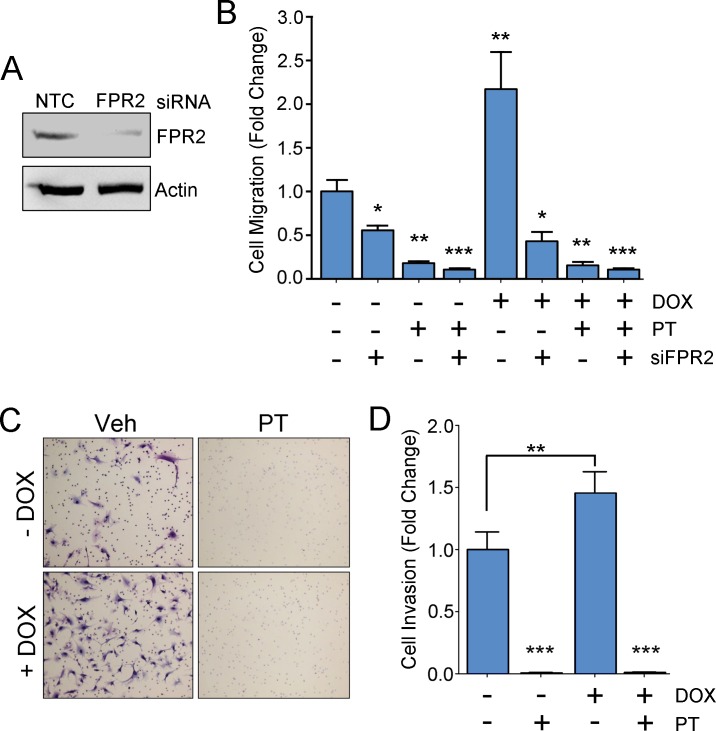
PT inhibits cell migration and invasion in HGG cells in which EGFRvIII expression is neutralized. (A) Immunoblot analysis of U251vIII cells transfected with FPR2 siRNA or NTC siRNA. Actin is shown as a loading control. (B) U251vIII cells, which were treated with doxycycline or vehicle, were transfected with FPR2-specific or NTC siRNA. PT (0.1 μg/mL) was added to the Transwells as indicated. The cells were allowed to migrate for 18 h. Migration is shown relative to untreated controls (mean ± SEM; n = 3; ***p<0.001). (C) Representative images of the undersides of Transwell membranes in Matrigel invasion assays. The U251vIII cells were pre-treated with doxycycline (+Dox) for 5 days to reverse expression of EGFRvIII or with vehicle (-Dox) to sustain EGFRvIII expression. PT (1.0 μg/mL) or vehicle (Veh) was added to the Transwell chambers. 1% FBS was added to the lower chambers. (D) Analysis of Matrigel invasion assays. Invasion is presented relative to untreated control wells (mean ± SEM; n = 3; ***p<0.001).

We previously showed that neutralizing EGFRvIII expression in U251vIII cells, by treating these cells with doxycycline, activates uPAR-dependent cell-signaling and as a result, promotes cell migration [9, 10]. Because the PT-sensitive GPCR, FPR2, is an essential co-receptor for uPAR-dependent cell-signaling [11, 12], we tested the role of PT in inhibiting U251vIII cell migration, before and after doxycycline treatment. To study the role of the uPAR cell-signaling system in these cell migration studies, we silenced FPR2 with siRNA. Fig 2A shows that FPR2 gene-silencing in U251vIII cells substantially decreased FPR2 protein expression, as determined by immunoblot analysis.

Fig 2B shows that when U251vIII cells were treated with doxycycline to neutralize EGFRvIII expression, cell migration was increased more than 2-fold, confirming our previous report [9]. FPR2 gene-silencing decreased migration of EGFRvIII-expressing cells U251 cells by 45% and doxycycline-treated cells by greater than 80%. As a result, FPR2 gene-silencing effectively neutralized the increase in cell migration associated with doxycycline treatment. PT (0.1 μg/mL) also significantly inhibited migration of both EGFRvIII-expressing and doxycycline-treated U251vIII cells, reversing the increase in cell migration associated with doxycycline treatment. These results suggest that targeting FPR2 or treating cells with PT inhibits the effects of uPAR on HGG cell migration.

### PT Inhibits HGG Cell Invasion

Next, we studied the ability of HGG cells to invade Matrigel, a model multicomponent extracellular matrix [27], reconstituted in Transwells. Invasion of Matrigel by cancer cells is promoted by activation of cell-signaling factors such as ERK1/2 and Rac1 and by activation of cell-surface proteases [27, 28]. uPAR has been implicated in both of these processes [28, 29]. U251vIII cells were treated with doxycycline to neutralize EGFRvIII expression [10]. Control cells were not doxycycline-treated and thus, expressed EGFRvIII. Fig 2C shows for the first time, that neutralizing EGFRvIII expression in U251vIII cells significantly increases Matrigel invasion.

PT (1.0 μg/mL) almost entirely blocked Matrigel invasion by U251vIII cells. Identical results were obtained when the HGG cells were treated with doxycycline to neutralize EGFRvIII expression or with vehicle so that EGFRvIII expression was sustained.

### PT Does Not Regulate Survival or Proliferation of HGG Cells

**Fig 3:**
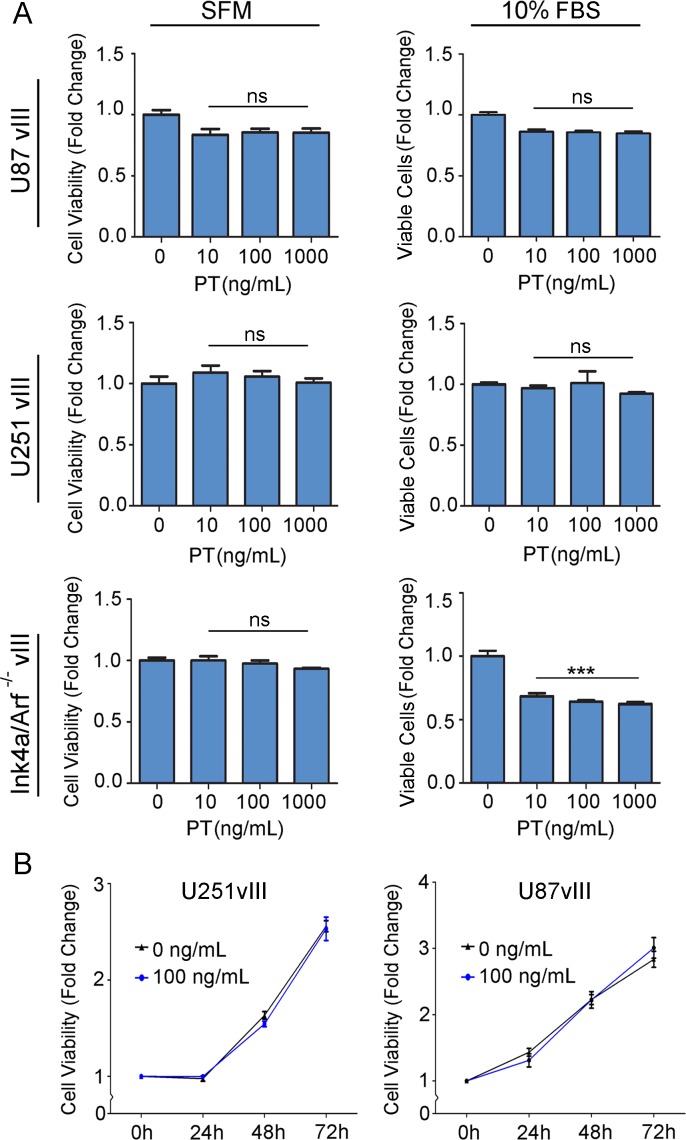
PT does not regulate survival or proliferation of HGG cells. (A) HGG cells were cultured in the presence of the indicated concentrations of PT in SFM or in medium supplemented with 10% FBS for 24 h. The number of viable cells was determined by WST-1 assay (mean ± SEM; n = 3; ***p<0.001). (B) U251vIII and U87vIII cells were cultured in the presence of 0.1 μg/mL PT or vehicle for up to 72 h. The number of viable cells was determined by WST-1 assay.

Changes in cell survival may influence cell migration results. We therefore conducted studies to examine HGG cell survival and proliferation. First, U87vIII cells were treated with 0.01–1.0 μg/mL PT for 24 h. The treatments were conducted in SFM to selectively examine cell survival and in the presence of 10% FBS to examine survival and proliferation. Fig 3A shows that PT failed to significantly affect the viable cell count under both sets of conditions. Equivalent results were obtained with U251vIII cells. PT did not alter Ink4a/Arf^-/-^vIII cell viability in SFM; however, a modest decrease in the viable cell count was noted when the Ink4a/Arf^-/-^vIII cells were treated with PT in the presence of 10% FBS.

To confirm that PT has no effect on HGG cell proliferation in U87vIII and U251vIII cells, we cultured these cells in 10% FBS-containing serum and 0.1 μg/mL PT or vehicle for up to 72 h. Fig 3B shows that cell growth was equivalent in the presence and absence of PT.

### Migration of HGG Cells Cultured in Neurospheres is Inhibited by PT

**Fig 4:**
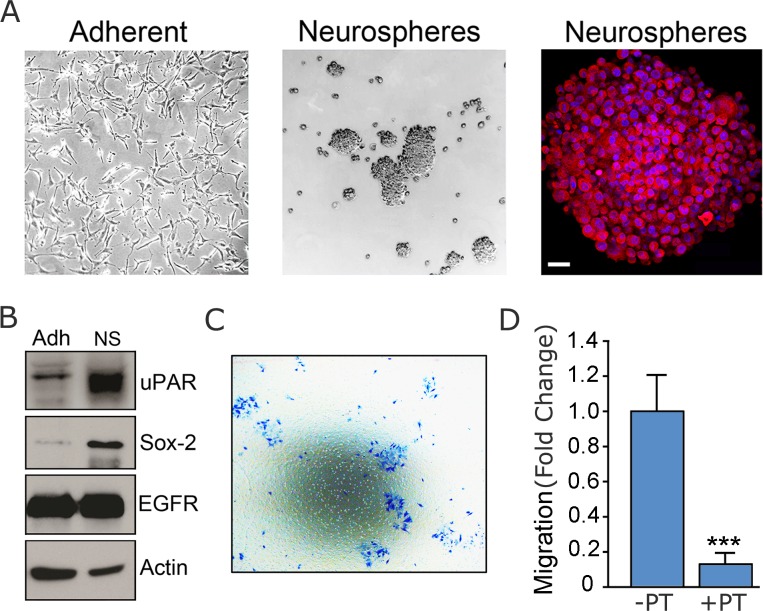
Migration of HGG cells isolated from neurospheres is inhibited by PT. (A) Phase contrast and confocal fluorescence imaging of U87vIII cells that were cultured either as (adherent) monolayers or as neurospheres. In the fluorescent confocal images, nuclei are stained with DAPI (blue). Actin cytoskeleton is stained with phalloidin (red). (B) Immunoblot analysis was performed to detect uPAR, Sox-2, EGFR and actin, as a control for load, in extracts of U87vIII cells, isolated from monolayer cultures (Adh) and neurospheres (NS). (C) Representative image of U87vIII cells, isolated from neurospheres, which migrated to the underside surface of Transwell membranes. (D) U87vIII neurospheres were isolated without disruption and added to upper chambers of Transwells. Cell migration proceeded for 4 h towards 1% FBS in the lower chamber. The medium was supplemented in both chambers with PT (0.5 μg/mL) or vehicle (-PT). Migration is shown relative to vehicle-treated control well (mean ± SEM; n = 3; ***p<0.001).

Brain tumor neurospheres are aggregates of HGG cells that form in suspension in SFM supplemented with defined growth factors [30, 31]. Neurospheres are thought to be enriched in multipotent tumor cells with stem cell-like properties, which promote tumorigenesis and are associated with increased invasiveness. Although most frequently isolated from intact tumors, HGG cells with stem cell-like activity have been identified in neurospheres formed by U87 cells [32]. We therefore compared U87vIII cells cultured in neurospheres versus cells cultured under standard monolayer conditions. Representative images of the cultures are shown in Fig 4A.

Because uPAR is a PT-target and major driver of HGG cell migration, we examined uPAR protein expression in U87vIII cells cultured in monolayers or neurospheres by immunoblot analysis. Fig 4B shows that uPAR protein was substantially increased in U87vIII cells in neurospheres. Sox-2 protein expression also was increased when U87vIII cells were cultured in neurospheres. Sox-2 is a previously described cancer stem cell biomarker [33].

Next, we studied the capacity of U87vIII cells, isolated from neurospheres, to migrate in Transwell systems. The neurosphere cultures were transferred to Transwells without disruption. Interestingly, Fig 4C shows that, despite the small size of the Transwell pores, many cells appeared to migrate across the membranes without losing cell contacts. Cells from neurospheres were recovered not only on the underside surface of the Transwell membranes, as is typical, but also in the lower reservoir, suggesting that the decreased capacity of these cells for adhesion was maintained.

We studied the effects of PT on migration of U87vIII cells, isolated from neurospheres, by determining the cell density on the underside of Transwell membranes. Fig 4D shows that PT (0.5 μg/mL) robustly inhibited migration of these cells.

### PT Suppresses Vimentin Expression in HGG Cells

**Fig 5:**
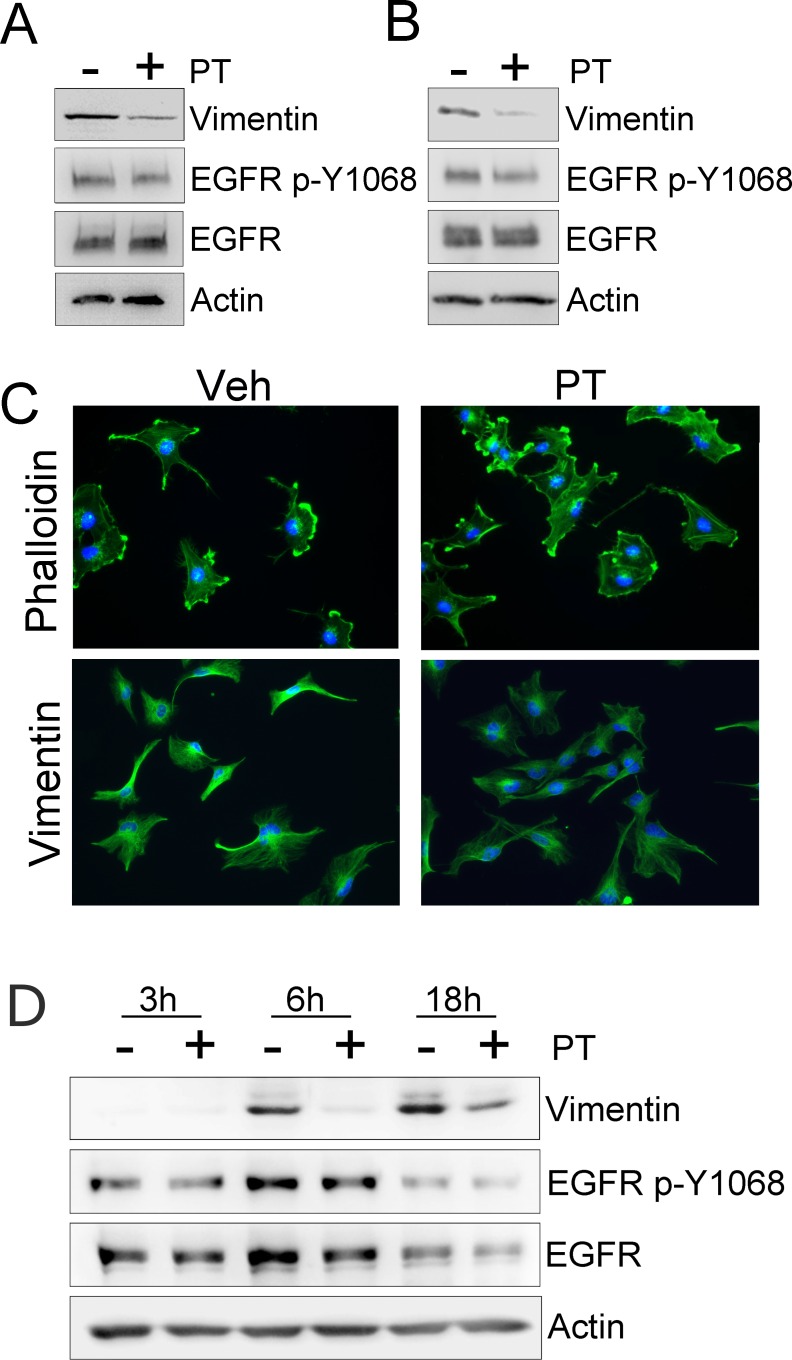
PT suppresses vimentin expression in HGG cells. (A) Immunoblot analysis to detect vimentin, EGFR phospho-Tyr-1068 (EGFR p-Y1068), total EGFR, and actin as a control for load in extracts of U251vIII cells that were treated with vehicle (-) or 1.0 μg/mL PT (+) for 24 h in 1% FBS. Cells were serum-starved for 24 h before treatment with PT. (B) Immunoblot analysis was performed as described in panel A to examine extracts of Ink4a/Arf^-/-^vIII astrocytes. (C) Fluorescent imaging of U251vIII cells that were treated for 24 h with 1 μg/mL PT or vehicle (Veh) in SFM. The two top panels show the actin cytoskeleton stained with Phalloidin (green) and the nuclei stained with DAPI (blue). The bottom panels show immunostaining to detect vimentin (green) and DAPI (blue). (D) Wild-type EGFR over-expressing U251 cells were treated with 15 ng/mL EGF in the presence (+) or absence (-) of 0.1 μg/mL PT for the indicated times, up to 18 h. Immunoblot analysis was performed to detect vimentin, EGFR phospho-Tyr-1068 (EGFR p-Y1068), total EGFR, and actin as a control for load.

Vimentin expression in HGG cells may indicate epithelial-mesenchymal transition (EMT) and be associated with increased rates of cell migration and worsened prognosis [21, 34]. Vimentin expression in HGG cells also may be associated with resistance to EGFR-targeted therapeutics [21]. Fig 5A shows that vimentin was detected by immunoblot analysis in extracts of U251vIII cells, which were isolated from monolayer cultures. When these cells were treated with 1.0 μg/mL PT for 24 h, vimentin expression was substantially decreased. Equivalent results were obtained when we treated Ink4a/Arf^-/-^vIII astrocytes with PT for 24 h (Fig 5B). IF microscopy was performed to examine U251vIII cells that were treated with 1.0 μg/mL PT or vehicle. As shown in Fig 5C, PT uniformly affected the cells, causing what appeared to be a similar decrease in vimentin immunopositivity. No evidence of cell death was observed.

To further test the role of PT in suppressing vimentin expression, wild-type EGFR over-expressing U251 cells were treated with 15 ng/mL EGF in the presence or absence of 0.1 μg/mL PT for up to 18 h. In the absence of PT, EGF increased vimentin expression, which was apparent by immunoblot analysis by 6 h. PT delayed the appearance of vimentin in response to EGF and the total level of vimentin remained decreased even at 18 h.

## Discussion

This study was initiated because of prior work suggesting that, in HGGs, activation of uPAR-dependent cell-signaling may provide resistance to EGFR-targeting therapies and promote HGG cell migration [9, 10, 26]. A variety of strategies have been applied to target uPAR in therapeutics development or take advantage of the fact that many malignancies express uPAR at high levels whereas most normal tissues express very low levels of uPAR [35, 36]. A problem encountered in the design of uPAR-targeting drugs concerns its numerous interactions with extracellular matrix (ECM) and membrane proteins that may be important for uPAR activity [29, 37, 38]. Components of the uPAR-signaling system include urokinase-type plasminogen activator, vitronectin, receptor tyrosine kinases, integrins, and GPCRs. No matter how the uPAR cell-signaling protein complex assembles, signaling responses are entirely blocked by PT given the essential role of FPR2 [10, 11, 39].

Our study was designed to examine the effects of PT on HGG cell migration, invasion, survival and growth. Our data clearly indicate that PT is a robust and selective inhibitor of HGG cell migration and Matrigel invasion. We used Matrigel as a model ECM with the known limitation that ECM in the brain has a very unique composition that may select for distinct invasion properties in cancer cells [40]. PT had limited or no effects on HGG cell viability and proliferation *in vitro*. These results indicate that PT is highly selective in its effects on HGG cell migration and invasion.

Because numerous receptors are PT targets, it is unlikely that the robust effects of PT on HGG cell migration are attributable to a single receptor or signaling system. However, we did collect data suggesting that the uPAR signaling system may be a major PT target. In U251vIII cells, the increase in cell migration induced by neutralizing EGFRvIII expression, which was previously attributed to the uPAR signaling system [9], was entirely blocked by PT. In our previous study [9], our approach for demonstrating the role of the uPAR signaling system in promoting HGG cell migration was to silence uPAR gene expression. In this study, we silenced expression of a second key member of this signaling system, FPR2, and obtained confirmatory data regarding the role of this system in HGG cell migration. Although combining FPR2 gene-silencing and PT treatment was marginally more effective than PT treatment alone at inhibiting cell migration, this result was not statistically significant. Importantly, in the present study, we have shown for the first time that neutralizing EGFRvIII expression in U251vIII cells not only promotes cell migration but also significantly increases the capacity of the cells to invade Matrigel. Invasion of Matrigel by U251vIII cells was entirely blocked by PT.

The feasibility of testing new drugs to treat brain tumors depends largely on the ability of the drugs to penetrate the blood brain barrier. Aggressive brain tumors frequently show abnormalities in the structure of the blood-brain barrier, in association with tumor angiogenesis, which allows for increased penetration by larger proteins [41]. Evidence that PT may access HGGs in patients may be found in numerous studies focusing on the induction of experimental autoimmune encephalopathy (EAE) in rodents [42]. Although PT is undoubtedly pleiotropic in EAE, PT may actually increase the permeability of the blood-brain barrier [43, 44]. Such activity may be particularly important in increasing exposure of HGG cells to PT when the tumor cells have disseminated into normal brain parenchyma.

We studied the effects of PT on migration of HGG cells isolated from neurospheres mainly because these cells expressed increased levels of uPAR. The neurosphere culturing method is thought to increase the density of cells with stem cell-like properties and this occurs to some extent even when it is applied to cell lines such as U87 cells [30–32]. Cancer stem cells are rare cells within a tumor that have the capacity to self-renew and differentiate into the diverse phenotypes observed in the parent tumor [45]. Cells with the characteristics of stem cells have been identified in HGG and thought to demonstrate increased resistance to chemotherapy and radiotherapy [46–48]. The effectiveness of PT in inhibiting migration of HGG cells isolated from neurospheres may represent an important property if this drug is tested in *in vivo* model systems or considered as a candidate therapeutic.

A major challenge in the use of recently developed targeted therapeutics is the emergence of molecular changes within cancer cells that confer resistance [38, 49]. Our recent study showing that activation of uPAR-initiated cell-signaling in cancer cells treated with EGFR-targeting drugs is associated with an increase in cell migration highlighted the possibility that compensatory changes in antitumor drug-treated cancer cells may be deleterious with regard to prognosis [9]. The current study advances this principle in demonstrating that neutralizing EGFRvIII in HGG cells may promote cell invasion. Counterproductive effects of anticancer drugs on cancer cell physiology may be avoided by combination therapies that target the signaling systems, which are activated in a compensatory manner [38].

We analyzed vimentin expression as a specific biomarker of the diverse changes in gene expression that probably occur in HGG cells treated with PT. Vimentin protein levels were suppressed in PT-treated cells, a result that was consistent with the decrease in the capacity of these cells to migrate and invade [21]. The effects of PT on HGG cell migration, invasion, and vimentin expression, in the absence of substantial effects on HGG cell survival and proliferation, indicate specificity in the PT receptors and systems targeted. Furthermore, the selective effects of PT on cell migration suggest that off-target effects on non-tumor cells may by controllable, as indicated by previous studies [15–17]. We conclude that PT and/or newly developed drugs with overlapping targets merit further study as therapies for HGG.

## Conclusions

Inhibiting the rapid dissemination of HGG cells throughout normal brain parenchyma may represent an important advance in treating HGG. We have shown that PT is a robust inhibitor of HGG cell migration, invasion through Matrigel, and vimentin expression. PT targets HGG cells maintained in monolayer culture and also cells cultured in neuropheres. The selective effects of PT on migration and invasion, without effects on cancer cell survival and proliferation, suggest that this agent may confine HGG tumor cells within manageable surgical or radiotherapy margins.
